# Influence of Solidification Microsegregation on Heterogeneous Austenite Grain Growth in Nb–Ti Microalloyed Steel: Experimental Investigation and Phase-Field Simulation

**DOI:** 10.3390/ma19194107

**Published:** 2026-09-25

**Authors:** Haijie Wang, Jian Wu, Huasong Liu, Pu Wang, Jiaquan Zhang

**Affiliations:** 1 School of Metallurgical and Ecological Engineering, University of Science and Technology Beijing, No. 30 Xueyuan Road, Haidian District, Beijing 100083, China; d202310130@xs.ustb.edu.cn; 2 School of Materials and Physics Science, Nanchang University, No. 999 Xuefu Road, Honggutan District, Nanchang 330031, China; 17870141700@163.com; 3 Institute for Special Steels, Central Iron and Steel Research Institute (CISRI), No. 76 Xueyuan South Road, Haidian District, Beijing 100081, China; liuhuasong_ustb@163.com

**Keywords:** grain growth, mixed-grain structure, segregation, second-phase particles, phase-field simulation

## Abstract

To elucidate austenite grain coarsening and mixed-grain formation in continuously cast Nb–Ti microalloyed steel during high-temperature holding, high-temperature holding experiments, Clyne–Kurz microsegregation calculations, Thermo-Calc equilibrium precipitation analysis, Gladman pinning calculations, and multi-order-parameter phase-field simulations were combined to investigate the effect of precipitation differences between dendritic and interdendritic regions on heterogeneous austenite grain growth. The experimental results show that mixed-grain structures of varying severity are present after holding for 1 h at 1050–1300 °C. The austenite grains coarsen overall with increasing temperature, while pronounced coarsening of the initially fine-grained regions occurs above approximately 1200 °C. Microsegregation calculations suggest that Nb, Ti, and C are enriched in the interdendritic region, which may promote higher Nb(C,N) and TiN precipitation compared with the dendritic region. As temperature increases, grain-boundary mobility increases continuously. At 1150 °C, a considerable amount of precipitates remains in the interdendritic region, whereas pinning in the dendritic region is weaker, causing the difference in effective grain-boundary mobility between the two regions to increase. At higher temperatures, extensive dissolution of interdendritic Nb(C,N) reduces the regional difference in pinning and, consequently, the difference in effective grain-boundary mobility. The phase-field simulations capture the general characteristics of heterogeneous austenite grain-growth behavior and provide mechanistic insight into the influence of spatially varying effective grain-boundary mobility: at 1050 °C, low grain-boundary mobility and strong pinning result in slow grain coarsening; near 1150 °C, preferential growth in the dendritic region while the interdendritic region remains pinned produces the highest mixed-grain severity; at 1300 °C, extensive precipitate dissolution causes pronounced overall grain coarsening while reducing the regional grain-size contrast. The experimental observations were further interpreted using microsegregation and precipitation calculations to reveal the possible relationship between solidification-induced compositional heterogeneity and heterogeneous grain growth. These results indicate that local variations in effective grain-boundary mobility associated with solidification microsegregation are an important factor influencing the formation of mixed-grain structures.

## 1. Introduction

Nb–Ti microalloyed steels can effectively suppress austenite grain-boundary migration and refine the microstructure through the formation of second-phase particles such as Nb(C,N) and TiN, and are therefore widely used in structural components requiring high strength and high fatigue resistance [1,2]. However, these steels commonly experience relatively high temperatures and prolonged holding during reheating, carburizing, or austenitizing. As temperature increases, grain-boundary mobility increases, while second-phase particles progressively coarsen or dissolve, making rapid austenite grain growth more likely and eventually promoting mixed-grain structures in which coarse- and fine-grained regions coexist. Such grain-size heterogeneity may lead to nonuniform local transformation behavior, hardenability, and mechanical properties. Therefore, clarifying the mechanism responsible for heterogeneous austenite grain growth during high-temperature holding is of considerable importance [2,3].

Austenite grain growth is jointly controlled by grain-boundary mobility and second-phase particle pinning. Fine, dispersed Nb(C,N) and TiN particles can restrict grain-boundary migration through Zener pinning; when these precipitates coarsen or dissolve, the pinning effect weakens and the grain-growth rate increases [2]. In continuously cast slabs, however, precipitates are not uniformly distributed. During solidification, elements such as Nb, Ti, and C tend to enrich in interdendritic regions, producing different local compositions and precipitation states in dendritic and interdendritic regions [4,5,6]. Previous studies have shown that, during continuous-casting solidification, Nb, Ti, and C are redistributed as the solid–liquid interface advances and become enriched to varying degrees in the residual interdendritic liquid that solidifies last. Kunze et al. [7] investigated Ti microsegregation and TiN precipitation during dendritic solidification of low-alloy steel and confirmed that interdendritic solute enrichment significantly promotes TiN formation. Roy et al. [6] further characterized the spatial distribution of precipitates in a continuously cast HSLA steel containing Nb, Ti, and V, and identified precipitate-rich and precipitate-depleted regions corresponding to the dendritic structure; the number density and volume fraction of Nb–Ti complex carbonitrides were markedly higher in the enriched regions. Zhang and Strangwood [8] noted that, although high-temperature diffusion after solidification can reduce microsegregation to some extent, compositional variations on the dendritic scale cannot be completely eliminated during conventional cooling. Consequently, before reheating, continuously cast slabs already exhibit structural heterogeneity characterized by interdendritic solute enrichment and relative solute depletion in dendritic regions, which is further translated into a spatially nonuniform distribution of second-phase particles such as Nb(C,N) and TiN [9]. This precipitate heterogeneity directly produces different grain-boundary pinning capacities in dendritic and interdendritic regions. Based on reheating experiments at 1050–1225 °C on continuously cast slabs with different microalloying systems, together with Thermo-Calc calculations, Chakrabarti et al. [5] showed that differences in precipitate stability temperatures between solute-enriched and solute-depleted regions are a major cause of bimodal austenite grain structures in Nb-microalloyed steels. Roy et al. [10] likewise found that precipitate-rich regions in Nb–Ti microalloyed steels can retain strong grain-boundary pinning for extended periods, whereas grains in adjacent precipitate-depleted regions coarsen markedly, producing coarse- and fine-grained regions correlated with the original dendritic segregation morphology. Wang et al. [11] observed in an as-cast Al–Nb steel that, during reheating at 1160–1200 °C, residual Nb-rich particles in interdendritic regions restricted local grain growth, whereas dendritic regions coarsened markedly after AlN dissolution, ultimately producing a banded bimodal grain structure. In addition, Chakrabarti et al. [12] showed that local variations in Nb content caused by solidification segregation can further modify subsequent recrystallization kinetics, allowing the original austenite mixed-grain structure to persist or become amplified during rolling. Thus, austenite mixed-grain formation is not governed solely by the average alloy composition, average precipitate content, or holding temperature; rather, it fundamentally arises from the spatially heterogeneous precipitate distribution induced by solidification microsegregation and the resulting local differences in grain-boundary mobility. Compared with previous studies that mainly correlated precipitate heterogeneity with final grain structures, this study further introduces regional differences in effective grain-boundary mobility into a phase-field framework. This approach enables the dynamic evolution of grain growth between solute-depleted and solute-enriched regions to be analyzed during holding, providing additional insight into the formation mechanism of mixed-grain structures. Previous experimental and modeling studies have further demonstrated that the evolution of Nb- and Ti-containing precipitates during reheating plays an important role in controlling austenite grain growth. With increasing temperature and holding time, Nb(C,N) particles progressively dissolve or coarsen, whereas TiN generally exhibits higher thermal stability. The associated changes in precipitate volume fraction, particle size, and number density modify the pinning pressure acting on austenite grain boundaries and may consequently trigger rapid or abnormal grain growth when the pinning force becomes insufficient. Previous grain-growth models have therefore incorporated the temperature- and time-dependent evolution of NbC/TiN precipitation into grain-growth calculations and demonstrated that consideration of precipitate evolution can substantially improve the description of austenite grain-growth kinetics [13,14]. These studies establish the importance of precipitation-controlled grain growth in Nb/Ti microalloyed steels. However, they generally describe the precipitate state and grain-growth response using an averaged material condition, whereas continuously cast steels inherently contain spatial variations in composition and precipitation state inherited from dendritic solidification.

Although previous experimental and modeling studies have established the important role of precipitation evolution in austenite grain growth, most investigations have focused on experimentally characterized grain size and precipitate states, averaged precipitation-controlled grain-growth kinetics, or limiting grain-size predictions. Such approaches can effectively describe the overall influence of precipitates on grain growth, but they do not readily capture the spatial difference in grain-boundary migration between dendritic and interdendritic regions or its continuous evolution during holding. The phase-field method describes polycrystalline grain growth and grain-boundary migration using order parameters, providing an effective framework for studying grain evolution under spatially heterogeneous pinning conditions [15,16,17,18,19]. Accordingly, this study uses a continuously cast Nb–Ti microalloyed steel as the model material. High-temperature holding experiments are used to characterize austenite grain evolution at different temperatures and holding times; the Clyne–Kurz model and Thermo-Calc are employed to calculate compositional and precipitation differences between dendritic and interdendritic regions; and second-phase particle pinning is further converted into a local effective grain-boundary mobility and incorporated into a multi-order-parameter phase-field model. The study focuses on establishing the intrinsic relationship among solidification-induced dendritic segregation, spatially heterogeneous precipitation, local grain-boundary mobility differences, and heterogeneous austenite grain growth, thereby providing a theoretical basis for optimizing high-temperature heat-treatment schedules and controlling grain-size uniformity in Nb–Ti microalloyed steels. Although phase-field models have been widely applied to describe grain growth kinetics and abnormal grain growth behavior, most previous studies assumed relatively homogeneous material conditions, such as uniform composition or uniform second-phase particle distributions. For continuously cast Nb–Ti microalloyed steels, however, dendritic segregation produces spatial variations in solute concentration and precipitate stability before reheating. These local differences may result in heterogeneous grain-boundary migration behavior during subsequent heat treatment. Therefore, a key scientific challenge is to establish the relationship between solidification-induced microsegregation, heterogeneous precipitation, local grain-boundary mobility variation, and mixed-grain formation. In this study, the phase-field model is coupled with regional precipitation calculations to introduce spatially varying effective grain-boundary mobility, thereby providing a new approach for describing heterogeneous austenite grain growth caused by solidification microsegregation.

## 2. Research Methods

### 2.1. Experimental Material and Heat-Treatment Procedure

The experimental material was sampled from a continuously cast Nb–Ti microalloyed steel slab produced by the Baosteel Steelmaking Plant (Shanghai, China); its chemical composition is listed in Table 1. Six specimens with cross-sectional dimensions of 20 mm × 20 mm were taken from the near-surface region of the slab (0–15 mm from the surface) and treated in a DC-B125/5 muffle furnace (DOTRUST, Beijing, China). The specimens were heated at approximately 10 °C/min in argon protective atmosphere, and the holding time was counted after the specimens reached the target temperature (1050, 1100, 1150, 1200, 1250, and 1300 °C, respectively) and the furnace temperature became stable. After holding for 1 h, the specimens were immediately quenched in water to preserve the prior-austenite grain-boundary morphology. To minimize the influence of high-temperature decarburization on the surface microstructure, the outermost 5 mm was removed, and the prior-austenite grain structure was examined at a depth of 10 mm from the original slab surface. After heat treatment, the specimens were ground and polished, and the polished surfaces were etched using a saturated aqueous picric-acid solution with an activating agent. All specimens were etched using the same saturated aqueous picric-acid solution containing the activating agent. The etching procedure was carefully controlled to obtain clear prior-austenite grain boundaries while avoiding over-etching. The same optical microscopy conditions were used for all specimens to ensure consistency in grain-boundary identification. Prior-austenite grain boundaries were observed and imaged using a Leica DM4 M optical microscope (Leica Microsystems GmbH, Wetzlar, Germany). The grain-boundary network in the micrographs was traced using AutoCAD 2021, and a MATLAB R2021a (The MathWorks, Inc., Natick, MA, USA) program was used for image processing and grain-size determination. The grain-size measurement procedure is illustrated in Figure 1. Because the objective of this study was to investigate the temperature dependence of heterogeneous grain growth, one specimen was prepared for each holding temperature. All specimens were extracted from the same slab region to minimize compositional and microstructural variations. The statistical reliability of grain evolution was evaluated by statistical analysis of multiple microscopic fields containing approximately 1000 grains for each condition. For each condition, several microscopic fields were randomly selected within the analyzed region. Areas containing only extremely coarse or fine grains were not intentionally selected. The measured fields were combined to obtain statistically representative grain-size distributions. It should be noted that segregation intensity and dendritic morphology may vary along the slab thickness due to differences in solidification conditions. Therefore, the selected near-surface region represents a characteristic segregated region for mechanism investigation, while further analysis of different slab positions is required for evaluating the general applicability of the proposed mechanism.

To characterize the austenite grain-size distribution, approximately 1000 grains were measured, and their areas were converted into equivalent circle diameters (ECDs). To minimize operator-related variation, all grain boundaries were manually traced by the same operator using consistent identification criteria. The subsequent grain recognition and size calculation were performed automatically using MATLAB R2021a. Considering the large number of analyzed grains, minor uncertainties associated with manual tracing are expected to have limited influence on the overall statistical distribution. The complete grain-size range was divided linearly into 15 equal-width ECD intervals, from which grain-size frequency distributions were constructed. The distributions were classified as unimodal or bimodal according to their peak characteristics. For microstructures exhibiting a distinct bimodal distribution, the method proposed by Chakrabarti et al. [4] was adopted to identify the fine-grain and coarse-grain peaks separately.

### 2.2. Thermodynamic Analysis and Phase-Field Simulation

#### 2.2.1. Selection of the Computational Domain and Establishment of the Microsegregation Model

Previous studies have shown that microsegregation in cast slabs causes a nonuniform precipitate distribution and directly affects the heterogeneous distribution of austenite grains after reheating [9]. Regions with lower solute contents contain fewer precipitates and therefore tend to form coarse grains, whereas solute-enriched regions contain more precipitates and tend to retain finer grains. To more clearly reveal the mixed-grain structure after reheating, a region with relatively severe segregation was selected for phase-field calculations so that the relationship between the degree of slab segregation and subsequent austenite coarsening or mixed-grain severity could be more effectively assessed. A 12.5 mm × 12.5 mm specimen located 10 mm beneath the slab surface was used to characterize the dendritic segregation morphology. After grinding and polishing, the specimen was etched with a saturated aqueous picric-acid solution containing an activating agent to reveal the dendritic structure, and the cross-sectional morphology was imaged. A 1000 μm × 1000 μm region exhibiting relatively severe segregation was then selected as the computational domain for phase-field simulation and thermodynamic analysis. The morphology of the segregated dendritic regions within the computational domain was traced in Photoshop. Microsegregation was calculated using the widely adopted Clyne–Kurz method [6,20,21,22]. The composition of the solute-depleted region (dendritic region) was taken as the liquid composition at a solid fraction fs of 0.05, whereas that of the solute-enriched region (interdendritic region) was taken as the liquid composition at fs = 0.95 [7]. The liquidus and solidus temperatures of the investigated steel were calculated using Thermo-Calc 2021a (Thermo-Calc Software AB, Solna, Sweden) [21]. The solidification-front temperature was calculated following the methods of Ma et al. [23] and Liu et al. [24], and the average cooling rate was estimated from the measured secondary dendrite arm spacing (SDAS) [25]. The resulting local elemental compositions of the dendritic and interdendritic regions are listed in Table 2. It should be noted that the local compositions calculated by the Clyne–Kurz model represent characteristic solute-enriched and solute-depleted conditions rather than directly measured chemical compositions. The subsequent thermal history may partially reduce segregation through diffusion. Therefore, the calculated compositions are mainly used to describe the relative difference in solute distribution between dendritic and interdendritic regions.

#### 2.2.2. Thermodynamic Analysis Method

Roy et al. [10] reported that, after reheating and holding for 1 h, the measured amount of second-phase precipitation in Nb-containing steels approaches the equilibrium value. Therefore, Thermo-Calc equilibrium calculations were used here to estimate the relative precipitation differences between dendritic and interdendritic regions. Nevertheless, the possible influence of precipitation/dissolution kinetics during heating should be considered when interpreting the calculated results. Thermo-Calc was therefore used to calculate the mass fractions of Nb(C,N), TiN, and AlN in the solute-enriched and solute-depleted regions, thereby obtaining the equilibrium distribution of second-phase particles in different regions as a function of temperature. The calculated second-phase particle volume fractions were then introduced into the Gladman pinning equation to evaluate their effects on austenite grain size in different regions. The conversion between precipitate mass fraction and volume fraction is given by Equation (1).

(1) $$V_{f}=\frac{m_{f}\cdot \rho_{b}}{\rho_{p}}$$

where *m_f_* is the mass fraction, *ρ_b_* is the density of the cast slab (7.8 g/cm^3^), and *ρ_p_* is the density of the second-phase particles. The densities of Nb(C,N) and TiN are taken as 8.15 and 5.4 g/cm^3^, respectively.

According to Gladman pinning theory [26], the average austenite grain size can be calculated using Equation (2).

(2) $$D_{\gamma }=\frac{\pi }{6}\left(\frac{3}{2}-\frac{2}{Z}\right)\left(\frac{d}{f_{v}}\right)$$

where *Z* is the heterogeneity factor, taken as 2; d is the average size of the pinning particles, in μm; and *f_v_* is the volume fraction of the pinning phase. Based on the experimental data of Roy et al. [10], the average TiN particle size increases from 100 to 120 nm over the range 1000–1250 °C, while the average Nb(C,N) particle size increases from 50 to 150 nm. In the present calculations, the average pinning-particle size at all temperatures is assumed to be 100 nm.

#### 2.2.3. Phase-Field Simulation Method

The phase-field method uses field variables to describe the geometrical morphology of complex grain structures and can simulate a variety of microstructural processes, including nucleation, growth, coarsening, and field-induced grain evolution. It has been widely applied to grain-growth processes and grain-growth kinetics. In this study, a phenomenological multi-order-parameter phase-field model was implemented in MATLAB R2021a to simulate austenite grain growth during reheating of the experimental steel. A set of phase-field variables, *η_1_(r,t), η_2_(r,t), …, η_p_(r,t)*, is used to distinguish grains with different crystallographic orientations. These order parameters are functions of spatial position and time. Inside grain i, η_i_ = 1 or −1 while all other order parameters are zero. Across a grain boundary, η_i_ varies continuously from 1 (or −1) to 0, while the order parameter associated with the adjacent grain changes from 0 to 1 or −1. The evolution of the phase-field variables is driven by a decrease in the total free energy of the system. The total free-energy functional can be expressed as [13,14,15,16]:

(3) $$F=\int_{V}\left\{\sum_{i=1}^{Q}\left(-\frac{1}{2}\alpha \eta_{i}^{2}+\frac{1}{4}\beta \eta_{i}^{4}\right)+\gamma \sum_{i=1}^{Q}\sum_{j\neq i}^{Q}\eta_{i}^{2}\eta_{j}^{2}+\sum_{i=1}^{Q}\frac{\kappa_{i}}{2}{\left|\nabla \eta_{i}\right|}^{2}\right\}dV$$

where *κ_i_* is the gradient-energy coefficient and Q is the total number of phase-field order parameters. The coefficients *α*, *β*, and *γ* are phenomenological parameters controlling the local free-energy landscape and interaction among different grain order parameters. In the present model, *α* = 1, *β* = 1, and *γ* = 1.5 were selected following previous multi-order-parameter grain-growth phase-field studies. These coefficients determine the mathematical characteristics of grain evolution, whereas the actual material-dependent grain-boundary behavior is introduced through the gradient-energy coefficient κ and interface kinetic coefficient L, which are related to grain-boundary energy, boundary width, and mobility. The evolution of each order parameter is described by the time-dependent Ginzburg–Landau (TDGL) equation [27]:

(4) $$\frac{\partial \eta_{i}}{\partial t}=-L_{i}\frac{\delta F}{\delta \eta_{i}},\;\;i=1,2,\ldots ,Q$$

where *L_i_* is the interface kinetic coefficient. Assuming isotropic mobility and gradient-energy coefficients, Equations (3) and (4) yield the governing equation for the evolution of the order parameters during grain growth:

(5) $$\frac{\partial \eta_{i}}{\partial t}=-L\left(-\alpha \eta_{i}+\beta \eta_{i}^{3}+2\gamma \eta_{i}\sum_{j\neq i}^{Q}\eta_{j}^{2}-\kappa \nabla^{2}\eta_{i}\right),\;\;i=1,2,\ldots ,Q$$

The model parameters, namely, the interface kinetic coefficient L and gradient-energy coefficient *κ*, are calculated as follows:

(6) $$\kappa =\frac{3}{4}\gamma_{GB}l_{GB}$$

(7) $$L=\frac{4}{3}\frac{M_{GB}}{l_{GB}}$$

where *γ_GB_* is the grain-boundary energy, *l_GB_* is the grain-boundary width, taken as 5 × 10^−4^ cm, and *M_GB_* is the grain-boundary mobility. The austenite grain-boundary mobility is a function of temperature and composition and is expressed by Equation (8) [28].

(8) $$M_{GB}=M_{0}\exp\left(-\frac{Q}{RT}\right)$$

where *M_0_* is the pre-exponential factor, *Q* is the activation energy for grain-boundary migration, *R* is the universal gas constant, and *T* is the absolute temperature. The activation energy can be obtained by regression of experimental data, and its relationship with the carbon equivalent is given by [29]:

(9) $$Q=167,686+40,562C_{p}$$

(10) $$C_{p}=C-0.14Si+0.04Mn$$

For Nb–Ti microalloyed steel, the grain-boundary mobility and grain-boundary energy at 1050 °C can be taken as 0.71 × 10^−4^ cm^4^/(J·s) and 0.7 × 10^−4^ J/cm^2^, respectively [29]. The grain-boundary mobility parameters adopted here were obtained from a Nb–Ti microalloyed steel system reported in the literature, which provides a suitable reference for the present material. However, these parameters should be considered representative kinetic values rather than exact intrinsic values for the investigated steel because grain-boundary migration can also be affected by local composition, precipitate state, and microstructural conditions. Using Equations (8)–(10), the grain-boundary mobilities at different temperatures were calculated as listed in Table 3, while the grain-boundary energy was treated as a constant. In this model, grain-boundary energy was assumed to be constant over the investigated temperature range. Although grain-boundary energy may vary with temperature and local composition, this effect is considered secondary compared with the strong temperature dependence of grain-boundary mobility. Therefore, the constant grain-boundary energy assumption was adopted to simplify the model while focusing on the influence of spatially heterogeneous precipitation and effective mobility variation. 

To account for the resistance of second-phase particles to austenite grain-boundary migration, the effective grain-boundary mobility model proposed by Tonks et al. [30] was adopted. In this model, the pinning effect of second-phase particles is represented as a reduction in grain-boundary mobility:

(11) $$M_{eff}=M_{GB}\left(1-\frac{1}{1+3{\left(\frac{\delta_{r}}{\bar{r}}\right)}^{2}}\frac{3}{4}\frac{WRf_{v}d}{\bar{r}}\right)$$

where *M_eff_* is the effective grain-boundary mobility after accounting for particle pinning; *δ_r_* and $\bar{r}$ are the standard deviation and mean of the particle-size distribution, respectively; W is a weighting constant; *R* is an exponent characterizing the spatial distribution of particles; *f_v_* is the particle volume fraction; and d is the average grain size. Assuming that the precipitate particles are uniformly distributed and neglecting the particle-size distribution, *W* = 1.1, *R* = 1, and *δ_r_* = 0. Equation (11) can then be simplified to:

(12) $$M_{eff}=M_{GB}\left(1-\frac{3.3}{4}\frac{f_{v}d}{\bar{r}}\right)$$

Based on the different second-phase particle volume fractions in the dendritic and interdendritic regions, Equation (12) was used to calculate the corresponding effective grain-boundary mobilities, *M_eff_*, for the two regions. In the phase-field simulations, the appropriate *M_eff_* value was assigned according to the spatial distribution of the dendritic and interdendritic regions, thereby representing local variations in grain-boundary migration capability caused by solidification microsegregation.

The phase-field computational domain was 1000 μm × 1000 μm, with a grid spacing of 1 μm and a time step of 0.04 s. The model was solved by the finite-difference method with periodic boundary conditions and implemented in MATLAB R2021a. Considering computational efficiency while minimizing artificial grain coalescence, the total number of order parameters was set to 48. The computational parameters were selected based on the balance between spatial resolution and computational efficiency. Because the characteristic grain size after reheating was much larger than the grid spacing, a 1 μm mesh was considered sufficient to describe grain-boundary evolution. The use of 48 order parameters allowed multiple grains to coexist while avoiding excessive computational cost. Nevertheless, systematic sensitivity analysis involving different mesh resolutions and order-parameter numbers would further improve the quantitative reliability of the model. During initialization, each nodal order parameter was assigned a random value between −0.001 and 0.001. Because the initial order parameters were randomly assigned, different random configurations may lead to variations in local grain morphology. However, this study focuses on the statistical evolution of heterogeneous grain growth induced by spatially varying effective grain-boundary mobility rather than the exact evolution of a specific initial grain structure. Multiple random initialization simulations would further improve the statistical validation of the phase-field results. For efficient matrix operations, the Laplacian operator was discretized using a five-point stencil. The time derivative was discretized using a backward-difference scheme:

(13) $$\frac{\partial \eta_{i}\left(x,t\right)}{\partial t}=\frac{\eta_{i}(x,t+\Delta t)-\eta_{i}(x,t)}{\Delta t},\;\;i=1,2,\ldots ,Q$$

## 3. Results and Discussion

### 3.1. Experimental Results

Figure 2 shows the austenite grain structures after holding the specimens for 1 h at different temperatures. The average austenite grain size increases progressively with increasing holding temperature, while coarse- and fine-grained regions coexist to varying degrees under all conditions. At 1050 °C, a certain degree of grain-size heterogeneity is already evident. With increasing temperature, the contrast between the coarse- and fine-grained regions becomes progressively more pronounced and reaches its maximum near 1150 °C. When the temperature is further increased to 1200 °C and above, grains in the initially fine-grained regions begin to coarsen markedly, resulting in a gradual reduction in the grain-size contrast. At 1300 °C, although coarse- and fine-grained regions can still be distinguished, the overall grain size is substantially larger and the degree of grain-size heterogeneity is lower than that observed near 1150 °C.

To further characterize the grain-size distribution at different temperatures, grains from multiple fields of view were measured and the resulting frequency distributions are presented in Figure 3. At the same holding time, the average grain size increases from 59.28 μm at 1050 °C to 159.46 μm at 1300 °C. The heterogeneous grain-growth behavior was evaluated based on statistical grain-size distributions obtained from approximately 1000 measured grains for each condition. These distributions provide quantitative evidence for the coexistence of coarse- and fine-grain populations at different holding temperatures. A further quantitative evaluation of mixed-grain severity and its relationship with effective grain-boundary mobility is discussed in Section 3.4. Combined with Figure 2, the grain-size frequency distributions provide quantitative evidence for varying degrees of coarse- and fine-grain coexistence throughout the range 1050–1300 °C, corresponding to grain-size distributions with different degrees of bimodality. Because the coarse grains are concentrated in localized regions and are larger but less numerous, the second peak corresponding to the coarse-grain population is generally lower than the fine-grain peak in number-based frequency distributions, and at some temperatures it appears only as a weak shoulder or peak. This behavior demonstrates that coarse- and fine-grain coexistence is spatially heterogeneous and is likely related to local variations in precipitation state and grain-boundary migration capability induced by dendritic segregation. The underlying mechanism is analyzed below by combining thermodynamic calculations and phase-field simulations.

### 3.2. Thermodynamic Calculation Results

Using the compositions of the segregated regions listed in Table 2, Thermo-Calc was employed to calculate the equilibrium precipitation of each phase in the solute-enriched and solute-depleted regions as a function of temperature, as shown in Figure 4. Pronounced differences in precipitation behavior are observed between the interdendritic and dendritic regions because of microsegregation. The solute-enriched interdendritic region contains larger amounts of TiN and Nb(C,N) than the solute-depleted region, whereas the AlN content is higher in the latter. Among the calculated precipitate phases, AlN exhibits a much lower fraction than Nb(C,N) and TiN. Therefore, Nb(C,N) and TiN were considered the dominant contributors to grain-boundary pinning in the present Gladman analysis. Although AlN may provide an additional pinning contribution, particularly in the solute-depleted region, its relatively small fraction is expected to have a limited influence on the comparison between dendritic and interdendritic regions. Accordingly, the heterogeneous austenite grain growth is considered to be primarily associated with the spatially nonuniform precipitation of Nb(C,N) and TiN. In the interdendritic region, the amount of Nb(C,N) decreases rapidly near 1200 °C and approaches complete dissolution at approximately 1250 °C. The rapid decrease in Nb(C,N) precipitation near 1200 °C substantially weakens grain-boundary pinning in the interdendritic region, which is consistent with the pronounced coarsening of the initially fine-grained regions observed at 1200 °C and above [31]. The assumed particle size may influence the absolute values predicted by the Gladman model because larger particles provide weaker pinning. However, this study mainly focuses on the relative difference in precipitation-induced pinning between dendritic and interdendritic regions. Therefore, the constant particle size assumption is expected to affect the quantitative grain-size prediction but has limited influence on the interpretation of the segregation-induced difference in grain-growth behavior.

The solute-enriched and solute-depleted regions were identified based on the dendritic segregation morphology revealed by metallographic etching. The interdendritic regions corresponding to the segregation-enriched zones were regarded as solute-enriched regions, whereas the dendritic cores were regarded as relatively solute-depleted regions. Grain sizes within these characteristic regions were then measured separately and compared with the corresponding Gladman-model predictions. The results are presented in Figure 5. Figure 5 shows the precipitate volume fractions and austenite grain sizes predicted by the Gladman model for the dendritic and interdendritic regions when only Nb(C,N) and TiN are considered. The discrepancy between the Gladman prediction and experimental grain size in the solute-depleted region may originate from the simplified assumptions of the model. The Gladman equation describes the limiting grain size controlled by particle pinning, whereas the experimentally observed grain size is also influenced by grain-boundary mobility, initial grain structure, and grain-growth kinetics. Therefore, the Gladman calculation is mainly used to evaluate the relative difference in pinning capability between regions rather than accurately predict the final grain size. The precipitate mass fractions calculated by Thermo-Calc were first converted to volume fractions using Equation (1) and then substituted into the Gladman model, Equation (2), to calculate the austenite grain size in each region. According to the experimental data of Roy et al. [10], the average TiN particle size increases from approximately 100 to 120 nm between 1000 and 1250 °C, and the average Nb(C,N) particle size also increases markedly with temperature. Because this study focuses primarily on the difference in precipitate volume fraction caused by dendritic segregation, a constant average pinning-particle size of 100 nm was assumed for all temperatures and regions to simplify the calculations. Roy et al. [10] also reported that, after heating at 3 K/s and holding for 1 h, the actual amount of precipitation in the slab is close to the equilibrium value. Thus, based on the Thermo-Calc equilibrium precipitation results and the above particle-size assumption, the difference in second-phase particle pinning between the dendritic and interdendritic regions was calculated, as shown in Figure 5. For the solute-enriched region, the grain size predicted by the Gladman model agrees well with the measured data, whereas a relatively large discrepancy exists for the solute-depleted region. The calculations also indicate a pronounced grain-size difference between the solute-enriched and solute-depleted regions over the entire temperature range. In other words, reheating the slab to 1000–1300 °C is predicted to produce a pronounced mixed-grain structure throughout the range. Above 1200 °C, however, Nb(C,N) in the solute-enriched region approaches its complete dissolution temperature, causing the austenite grains in this region to grow rapidly and thereby reducing the mixed-grain severity. These trends are consistent with the experimental observations. This raises a further question: when increasing the holding temperature alone cannot eliminate the mixed-grain structure, how should mixed-grain severity or grain-size uniformity be evaluated? As shown in Figure 5, the difference predicted by the Gladman model varies little below 1300 °C but increases abnormally above 1300 °C, whereas experimental measurements are also strongly affected by the quality of grain-boundary etching and the number of grains included in the statistics. Therefore, to provide a more general analysis of the austenite grain structure and to examine the relationship between precipitate state and mixed-grain severity, phase-field simulations were used to calculate austenite grain growth during holding. The assumed particle size may influence the absolute values predicted by the Gladman model because larger particles provide weaker pinning. However, this study mainly focuses on the relative difference in precipitation-induced pinning between dendritic and interdendritic regions. Therefore, the constant particle size assumption is expected to affect the quantitative grain-size prediction but has limited influence on the interpretation of the segregation-induced difference in grain-growth behavior.

### 3.3. Phase-Field Simulation Results

The dendritic segregation morphology revealed by etching near the slab surface is shown in Figure 6. A 1000 μm × 1000 μm region with relatively severe segregation was selected as the computational domain for the phase-field simulations. The elemental contents in the interdendritic and dendritic regions were taken from Table 2. The phase-field simulation results are shown in Figure 7, Figure 8, Figure 9, Figure 10, Figure 11 and Figure 12. The selected simulation region exhibits relatively strong segregation and therefore represents a characteristic case for mechanism analysis. The predicted mixed-grain severity may be higher than that of regions with weaker segregation. Further simulations covering different segregation intensities are required to establish a quantitative relationship between segregation degree and grain-growth heterogeneity. The selected simulation temperatures represent three characteristic microstructural states: strong pinning-controlled growth at 1050 °C, maximum heterogeneous grain-growth tendency near 1150 °C, and precipitation-dissolution-dominated coarsening at 1300 °C. Therefore, these cases were chosen to reveal the underlying mechanism rather than provide a complete simulation of every experimental temperature. The phase-field simulations capture the general characteristics of heterogeneous austenite grain-growth behavior and provide mechanistic insight into the influence of spatially varying effective grain-boundary mobility. Because the effective mobility field is derived from coupled thermodynamic calculations, the agreement between simulation and experiment should be considered as consistency between the coupled model and experimental observations rather than as independent predictive validation.

Figure 7 and Figure 8 show the austenite grain morphologies and grain-size statistics at 1050 °C for different holding times. Overall, the austenite grains grow relatively slowly with increasing holding time, as shown in Figure 7a–f, indicating that the combined effects of low grain-boundary mobility and strong second-phase particle pinning restrict sustained grain-boundary migration. As the holding time increases from 10 to 60 min, the average grain size increases from 25.45 to 55.63 μm, corresponding to an increase of approximately 118.58%, which is reasonably consistent with the experimentally measured average grain size in Figure 2a. After holding for 60 min at 1050 °C, the fine-grain and coarse-grain peaks are located at approximately 50 and 90 μm, respectively, in agreement with Figure 3a, supporting the reliability of the simulation results.

At 1150 °C, grain growth becomes markedly faster, as shown in Figure 9a–f. Figure 10 summarizes the grain-size evolution at different holding times. As the holding time increases from 10 to 60 min, the average grain size increases from 35.73 to 74.91 μm, corresponding to an increase of approximately 109.7%, and the average grain size is in reasonable agreement with the experimental result in Figure 3c. With increasing holding time, some grains grow rapidly while a population of fine grains remains, producing a more pronounced mixed-grain structure. After holding for 60 min at 1150 °C, the fine-grain peak is approximately 60 μm and the coarse-grain peak is approximately 170 μm. These results indicate that, near 1150 °C, the intrinsic grain-boundary mobility has already increased substantially, while the interdendritic region remains strongly pinned by precipitates, resulting in a large difference in grain-growth rate between the two regions.

At 1300 °C, the austenite grains coarsen markedly with increasing holding time, as shown in Figure 11a–f. Figure 12 presents the grain-size statistics at different holding times. As the holding time increases from 10 to 60 min, the average austenite grain size increases from 58.80 to 155.61 μm, an increase of approximately 164.64%, which is substantially greater than those at 1050 and 1150 °C. After holding for 60 min at this high temperature, extensive dissolution of precipitates such as Nb(C,N) markedly reduces the resistance to grain-boundary migration, resulting in rapid and continuous austenite grain coarsening. Nevertheless, the microstructures and grain-size distributions show that the initially fine-grained regions also undergo rapid growth, shifting the overall distribution toward larger grain sizes. Consequently, although the average grain size is the largest, the mixed-grain severity is lower than that near 1150 °C. After holding for 60 min at 1300 °C, the fine-grain and coarse-grain peaks are approximately 150 and 230 μm, respectively, consistent with Figure 3f. The simulated average grain sizes obtained from the phase-field model are shown in Figure 3. These values were further compared with the experimentally measured grain sizes. Both experimental and simulated results exhibit the same temperature-dependent evolution tendency, namely, an increase in average grain size with increasing holding temperature. This consistency indicates that the phase-field model can capture the general characteristics of heterogeneous austenite grain growth induced by spatially varying effective grain-boundary mobility.

### 3.4. Quantitative Evaluation of Mixed-Grain Severity

To quantitatively evaluate mixed-grain severity at different temperatures, the area balance between coarse- and fine-grained regions was considered. When the areas occupied by coarse- and fine-grained regions are comparable, the mixed-grain structure is most pronounced; as the difference between these areas increases, the microstructure becomes increasingly dominated by one grain-size population and the mixed-grain severity decreases. Accordingly, the following mixed-grain index is defined:

(14) $$f_{mg}=1-\frac{\left|S_{cgr}-S_{fgr}\right|}{\left(S_{cgr}+S_{fgr}\right)}$$

where *f_mg_* is the mixed-grain index and *S_cgr_* and *S_fgr_* are the areas of the coarse-grain and fine-grain regions, respectively. A larger *f_mg_* indicates that the areas of the coarse- and fine-grained regions are more similar, corresponding to a greater degree of coarse/fine-grain coexistence and higher mixed-grain severity; conversely, a lower *f_mg_* indicates that one grain-size population increasingly dominates the microstructure. The mixed-grain indices at different temperatures are shown in Figure 13. With increasing holding temperature, *f_mg_* first increases and then decreases. Specifically, *f_mg_* is approximately 0.41 at 1050 °C, increases to approximately 0.59 at 1150 °C, and then decreases to approximately 0.25 at 1300 °C. Thus, the mixed-grain structure is most pronounced near 1150 °C, whereas at higher temperatures the fine-grained regions coarsen and the mixed-grain severity decreases substantially. This trend is broadly consistent with the experimental microstructures and grain-size distributions. It should be noted that the proposed *f_mg_* is not intended to replace conventional statistical parameters describing grain-size distributions. Instead, it specifically quantifies the spatial coexistence balance between coarse and fine-grain populations, which is the characteristic feature of mixed-grain structures investigated in this study. The mixed-grain index proposed here is intended to describe the spatial coexistence of coarse- and fine-grained regions rather than the statistical dispersion of grain size. Therefore, area balance was selected because the most pronounced mixed-grain structure occurs when coarse and fine-grain regions occupy comparable fractions. Other statistical descriptors, such as coefficient of variation or grain-size ratio, may provide complementary information but do not directly describe the spatial balance between two grain populations.

Figure 13 also shows the relative difference in effective grain-boundary mobility between the dendritic and interdendritic regions as a function of temperature. From 1050 to 1150 °C, the difference in effective grain-boundary mobility between the two regions progressively increases, whereas at higher temperatures it shows an overall decreasing trend. The temperature-dependent grain-growth behavior observed in this study is generally consistent with previous experimental and modeling studies of Nb/Ti microalloyed steels. These studies have shown that austenite grain growth during reheating is strongly dependent on the evolution of Nb(C,N) and TiN precipitates [13]. At relatively low temperatures, a high precipitate volume fraction provides strong grain-boundary pinning and restricts grain growth. With increasing temperature, dissolution and coarsening of Nb-containing precipitates reduce the pinning pressure, thereby increasing the tendency for rapid or abnormal austenite grain growth. TiN, owing to its relatively high thermal stability, can retain a pinning effect over a wider temperature range [13]. Previous coupled precipitation–grain-growth models have likewise demonstrated that simultaneous variations in precipitate size and volume fraction produce a strongly temperature-dependent pinning pressure and therefore substantially affect the limiting austenite grain size and grain-growth kinetics [14]. The present results extend these previous observations by considering the spatial heterogeneity of precipitation inherited from dendritic solidification. Rather than using only an averaged precipitate state, the present model distinguishes the solute-depleted dendritic region from the solute-enriched interdendritic region and assigns different calculated effective grain-boundary mobilities according to their precipitation states. Near 1150 °C, the dendritic region contains fewer precipitates and consequently exhibits weaker calculated pinning, whereas the interdendritic region still retains a considerable amount of Nb(C,N) and TiN and remains more strongly pinned. This produces the largest difference in effective grain-boundary migration capability and promotes the strongest coexistence of coarse and fine grains. When the temperature is increased further, extensive dissolution of Nb(C,N), particularly in the interdendritic region, reduces the difference in pinning between the two regions. Consequently, although the overall austenite grain size continues to increase, the grain-growth behavior of the two regions becomes more similar and the mixed-grain severity decreases. Therefore, the present results suggest that the intermediate-temperature maximum in mixed-grain severity can be understood as a spatial manifestation of precipitation-controlled grain growth under heterogeneous segregation conditions.

In summary, heterogeneous austenite grain growth in Nb–Ti microalloyed steel is controlled primarily by the difference in effective grain-boundary migration capability between dendritic and interdendritic regions. The nonmonotonic variation of mixed-grain severity with temperature is mainly associated with the competition between precipitation stability and grain-boundary migration capability. At 1050 °C, both dendritic and interdendritic regions contain relatively stable Nb(C,N) and TiN precipitates, resulting in strong particle pinning in both regions. Although the interdendritic region contains a higher amount of precipitates due to solute enrichment, the overall grain-boundary migration capability remains limited, and therefore the difference in grain-growth behavior between the two regions is relatively small. When the temperature increases to approximately 1150 °C, the system enters a transition regime of precipitate dissolution. Because the dendritic region contains lower concentrations of Nb, Ti, and C, Nb(C,N) and TiN particles become less stable and dissolve more readily. In contrast, the interdendritic region retains a higher precipitate stability due to solute enrichment. The resulting difference in precipitation-induced pinning pressure leads to the largest difference in effective grain-boundary migration capability between the two regions. Consequently, grains in the dendritic region undergo accelerated growth, while the interdendritic region remains relatively fine, producing the most pronounced mixed-grain structure. At temperatures above 1250 °C, extensive dissolution of Nb(C,N) and TiN occurs in both regions. Although the overall grain-growth rate increases due to enhanced atomic diffusion and grain-boundary mobility, the difference in pinning effect between dendritic and interdendritic regions becomes smaller. Therefore, the grain-size difference gradually decreases, and the mixed-grain severity is reduced. These results indicate that heterogeneous grain growth does not simply increase with temperature. Instead, it is controlled by the balance between precipitate stability and grain-boundary migration kinetics. The intermediate temperature range represents the condition where the spatial difference in effective grain-boundary migration capability is maximized. Thus, the mixed-grain severity first increases and then decreases in response to the temperature-dependent difference in effective grain-boundary migration capability between dendritic and interdendritic regions, reaching a maximum near 1150 °C. From an industrial perspective, the maximum mixed-grain severity near 1150 °C indicates a potential critical temperature region during reheating of continuously cast Nb–Ti steels. Long holding within this temperature range may amplify the inherited segregation-induced difference in grain growth behavior. Therefore, reheating schedules should consider not only the target austenitization temperature but also the heating path and residence time within this critical region. In practical processing, reducing severe solidification segregation, shortening the residence time around this temperature range, or applying appropriate homogenization treatments may help improve austenite grain-size uniformity. The regional difference in effective grain-boundary migration capability proposed in this study should be regarded as a mechanistic parameter rather than a directly measurable industrial parameter. In practical applications, this parameter may be estimated indirectly by combining segregation characterization, precipitation prediction, and grain-growth models. Such an approach may provide a potential method for evaluating the susceptibility of continuously cast microalloyed steels to heterogeneous grain growth during reheating.

## 4. Conclusions

This study investigated heterogeneous austenite grain growth in Nb–Ti microalloyed steel by combining high-temperature experiments, thermodynamic calculations, and phase-field simulations. The results provide insight into the possible relationship among solidification microsegregation, heterogeneous precipitation, regional differences in grain-boundary mobility, and mixed-grain formation. The main conclusions are as follows:

1. After holding for 1 h at 1050–1300 °C, the experimental steel exhibits coarse- and fine-grain coexistence to varying degrees. The austenite grains coarsen overall with increasing temperature. The mixed-grain severity is highest at 1150 °C; when the temperature is increased further above 1200 °C, the fine-grained regions also grow substantially and the mixed-grain severity decreases.
2. Clyne–Kurz calculations suggest that Nb and Ti are enriched in the interdendritic region. Thermo-Calc results further predict higher Nb(C,N) and TiN precipitation in this region, which may contribute to stronger calculated grain-boundary pinning. The amount of Nb(C,N) decreases rapidly near 1200 °C and approaches complete dissolution at approximately 1250 °C.
3. The precipitation difference caused by solidification microsegregation can be translated into a difference in effective grain-boundary mobility between dendritic and interdendritic regions, which is a key factor controlling heterogeneous austenite grain growth. The phase-field simulations reproduce the experimentally observed overall coarsening and nonmonotonic variation in mixed-grain severity with increasing temperature.
4. At 1050 °C, low intrinsic mobility together with stable precipitates suppresses grain growth. Near 1150 °C, migration accelerates in the dendritic region while the interdendritic region remains pinned, producing the largest regional difference in grain-growth rate and the highest mixed-grain severity. At 1300 °C, extensive precipitate dissolution leads to the most pronounced overall grain coarsening, but the grain-size contrast between coarse and fine regions decreases.

## Figures and Tables

**Figure 1:**
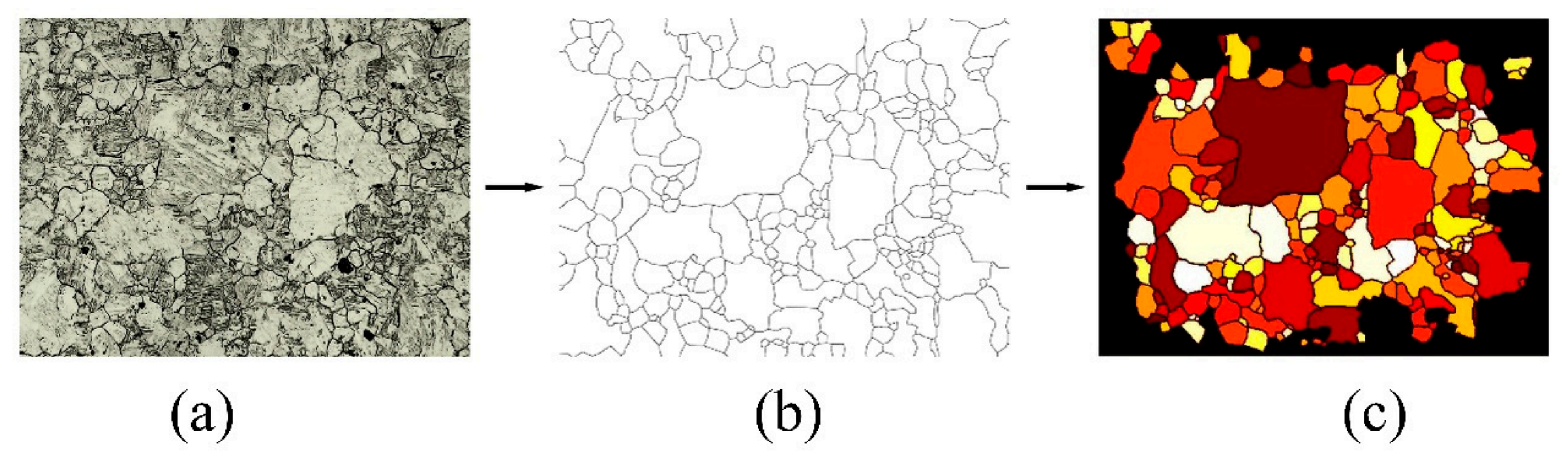
Schematic of the grain-size identification procedure: (**a**) optical micrograph; (**b**) grain boundaries traced in AutoCAD; (**c**) grain identification using MATLAB R2021a.

**Figure 2:**
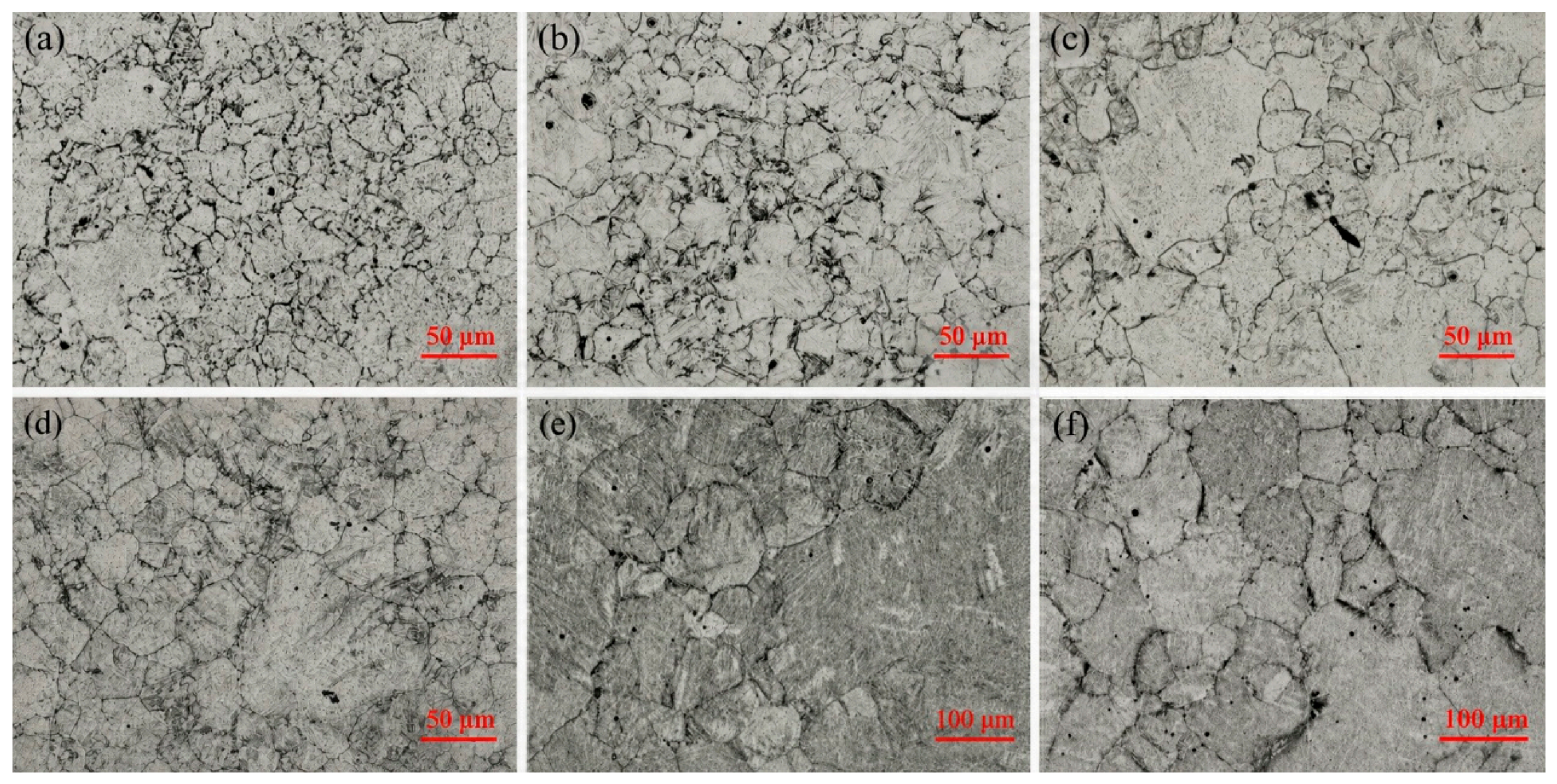
Austenite grain of the continuously cast slab after holding for 1 h at different temperatures: (**a**) 1050 °C; (**b**) 1100 °C; (**c**) 1150 °C; (**d**) 1200 °C; (**e**) 1250 °C; (**f**) 1300 °C.

**Figure 3:**
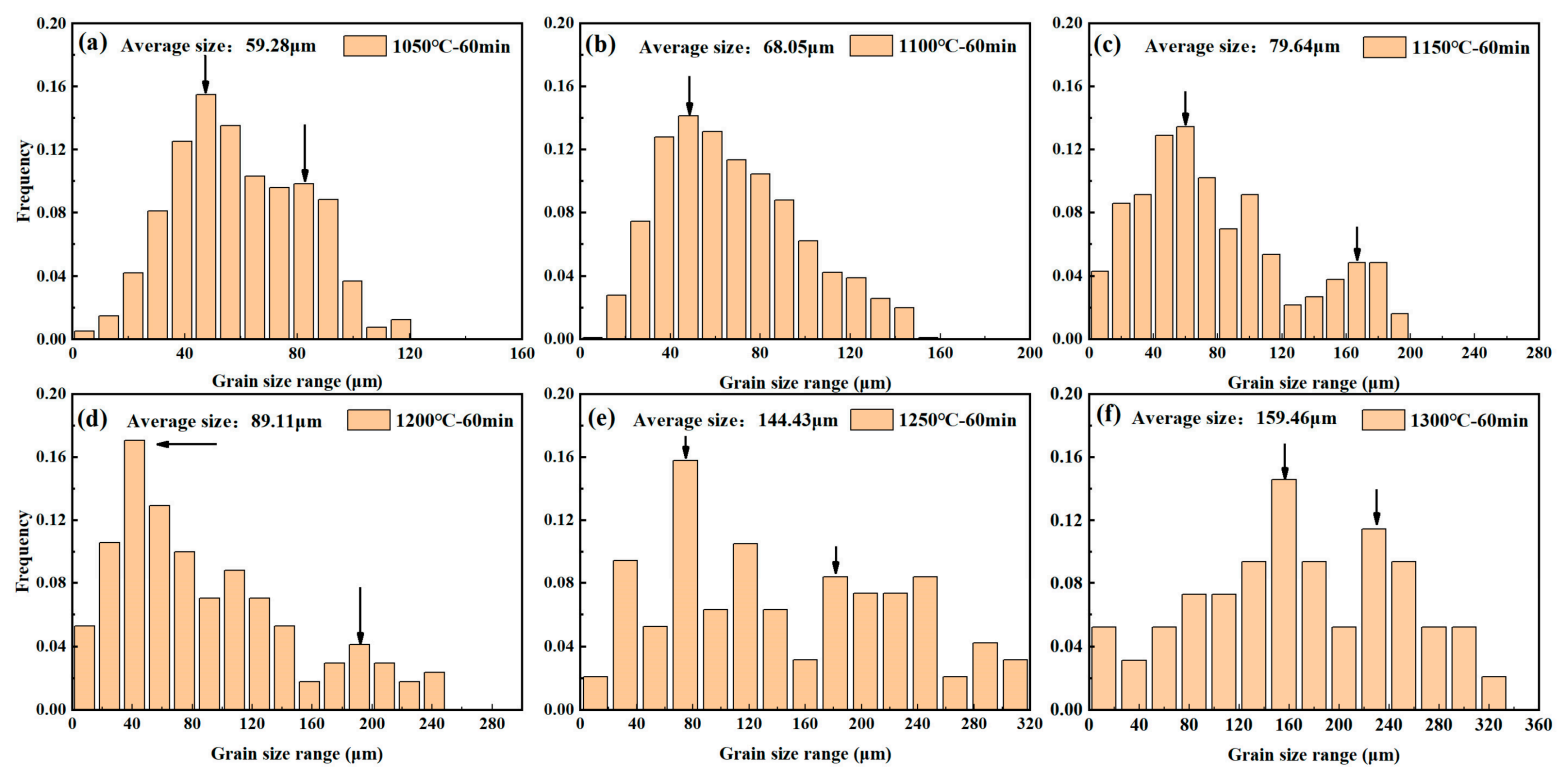
Austenite grain-size frequency distributions after holding for 1 h at different temperatures: (**a**) 1050 °C; (**b**) 1100 °C; (**c**) 1150 °C; (**d**) 1200 °C; (**e**) 1250 °C; (**f**) 1300 °C. (In the figure, the first arrow indicates the fine-grain peak, and the second arrow indicates the coarse-grain peak. the same applies below.)

**Figure 4:**
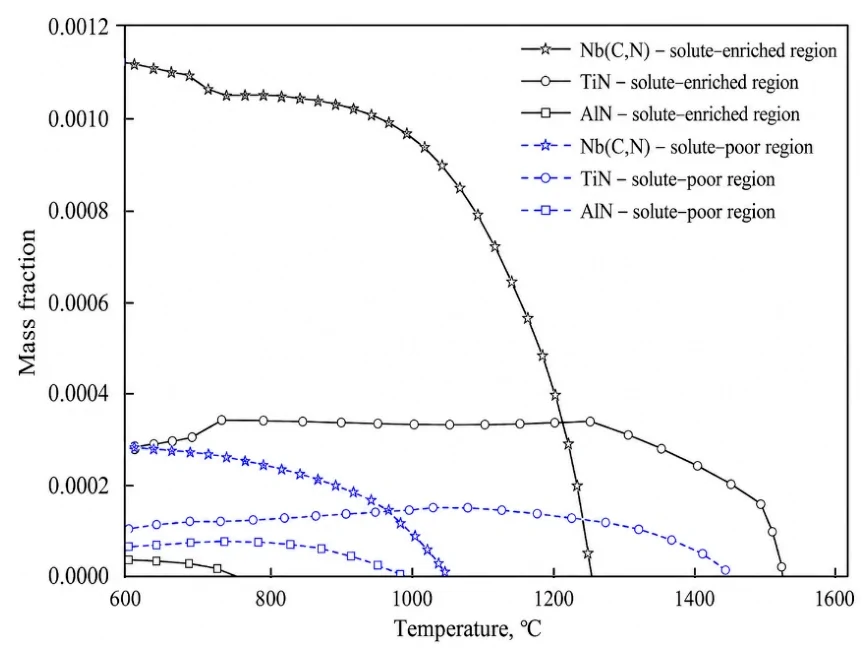
Calculated equilibrium precipitate fractions in solute-enriched and solute-depleted regions.

**Figure 5:**
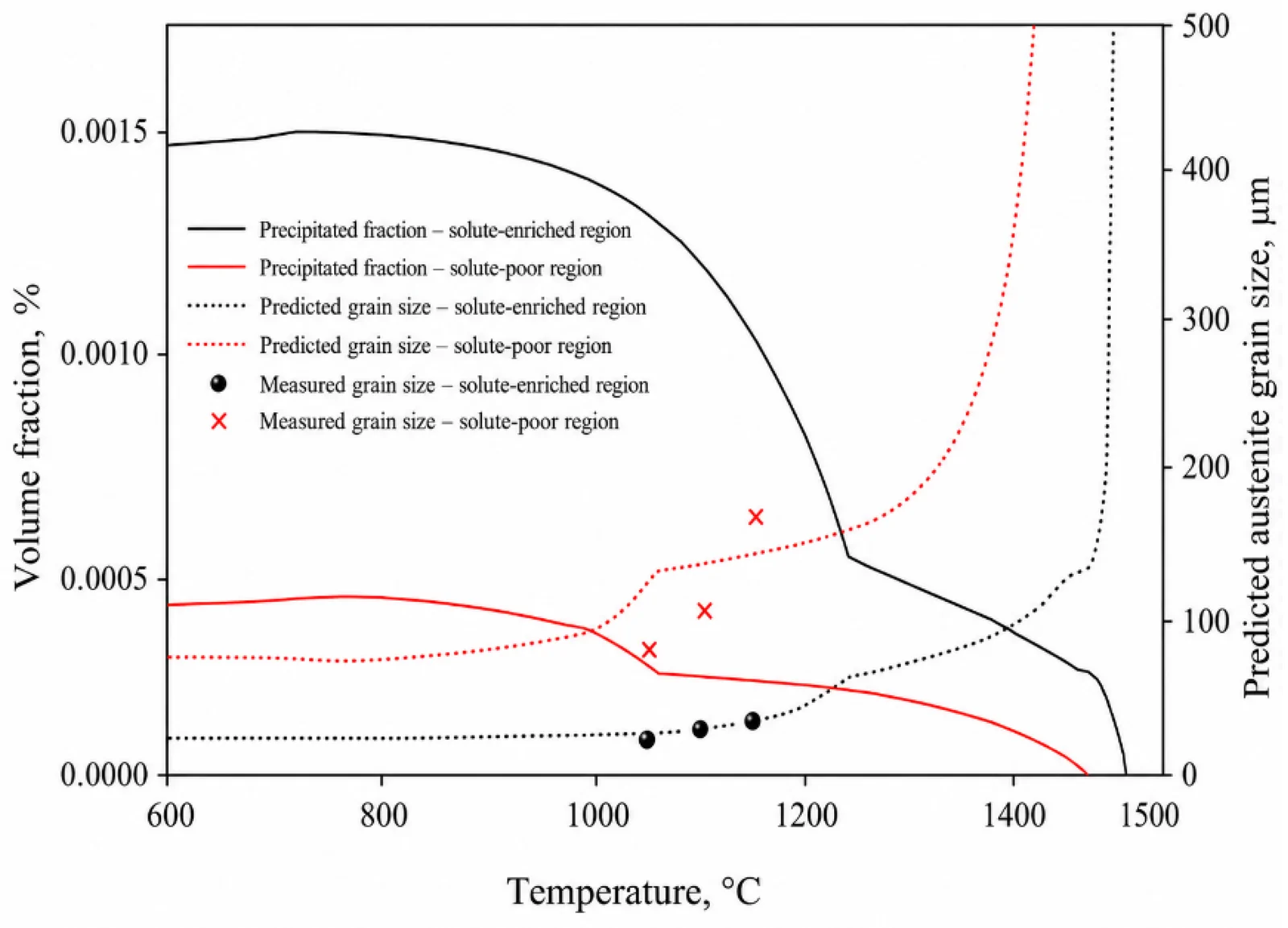
Temperature dependence of precipitate fraction and predicted austenite grain size in different regions.

**Figure 6:**
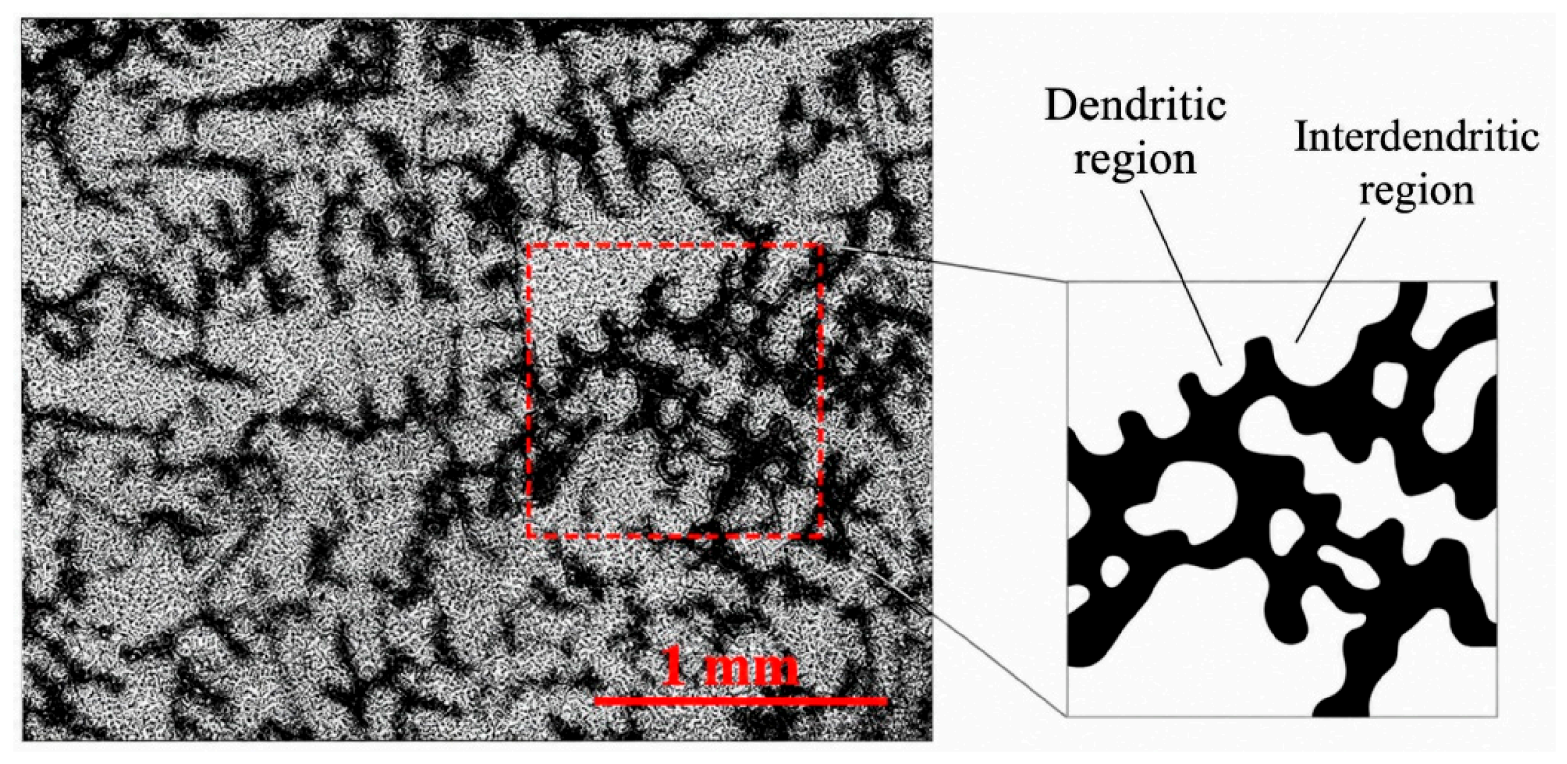
Selection of the computational domain and division of characteristic regions.

**Figure 7:**
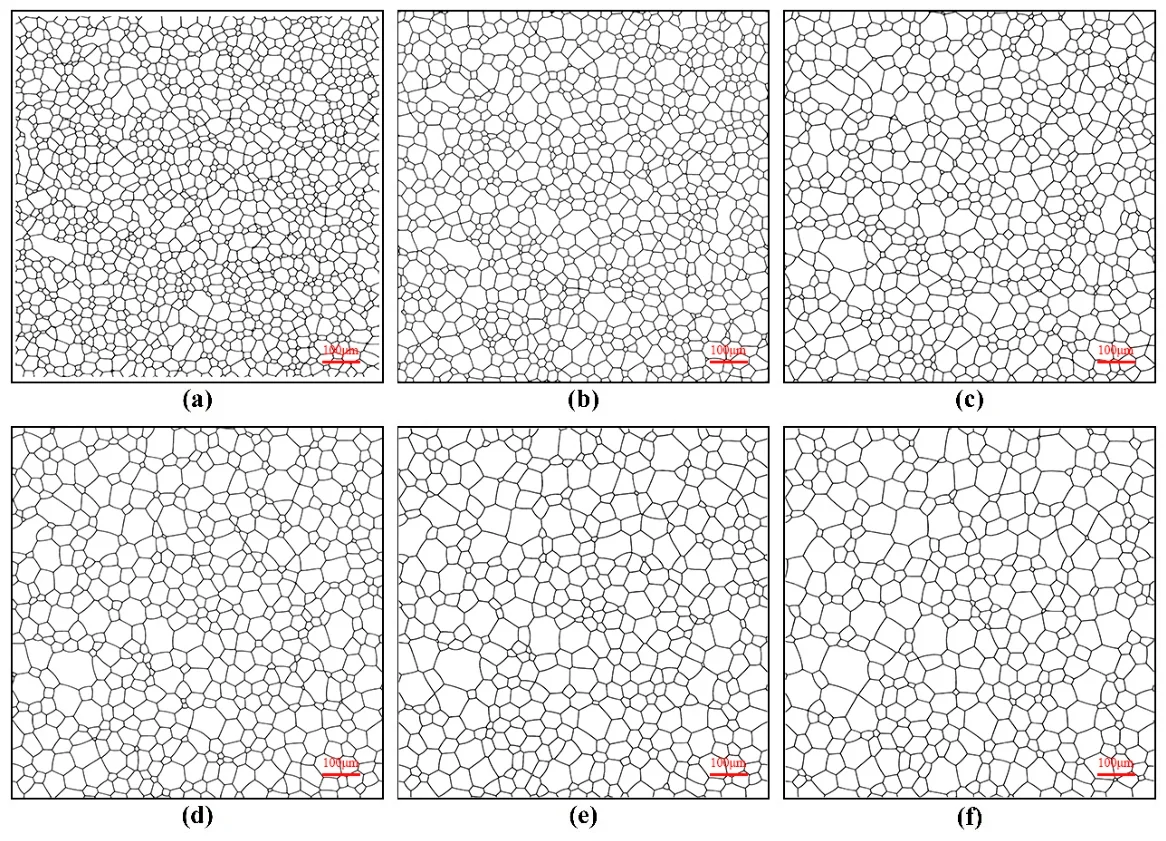
Evolution of the austenite grain structure during holding at 1050 °C: (**a**–**f**) holding times of 10, 20, 30, 40, 50, and 60 min, respectively.

**Figure 8:**
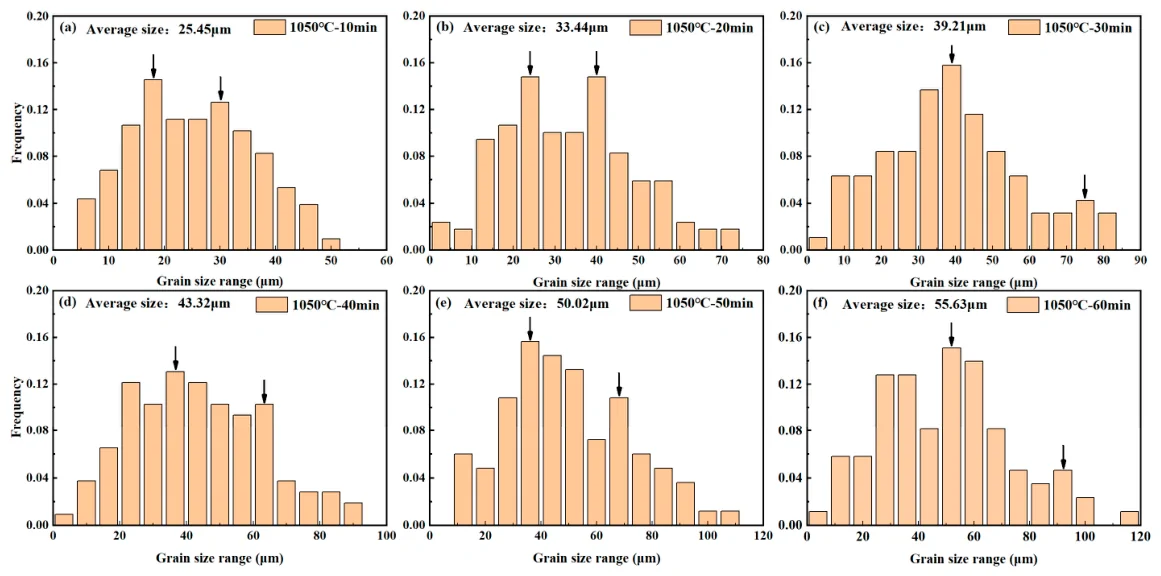
Simulated grain-size frequency distributions after different holding times at 1050 °C: (**a**) 10 min; (**b**) 20 min; (**c**) 30 min; (**d**) 40 min; (**e**) 50 min; (**f**) 60 min.

**Figure 9:**
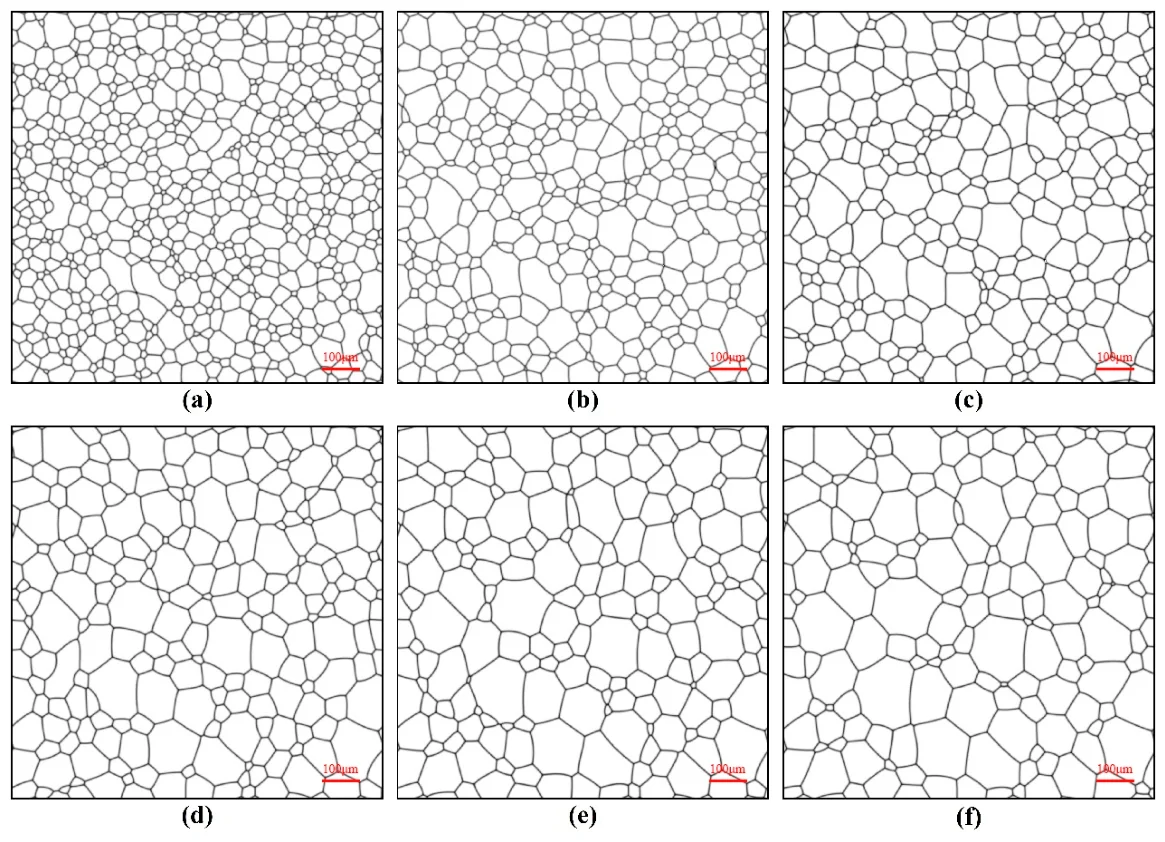
Evolution of the austenite grain structure during holding at 1150 °C: (**a**–**f**) holding times of 10, 20, 30, 40, 50, and 60 min, respectively.

**Figure 10:**
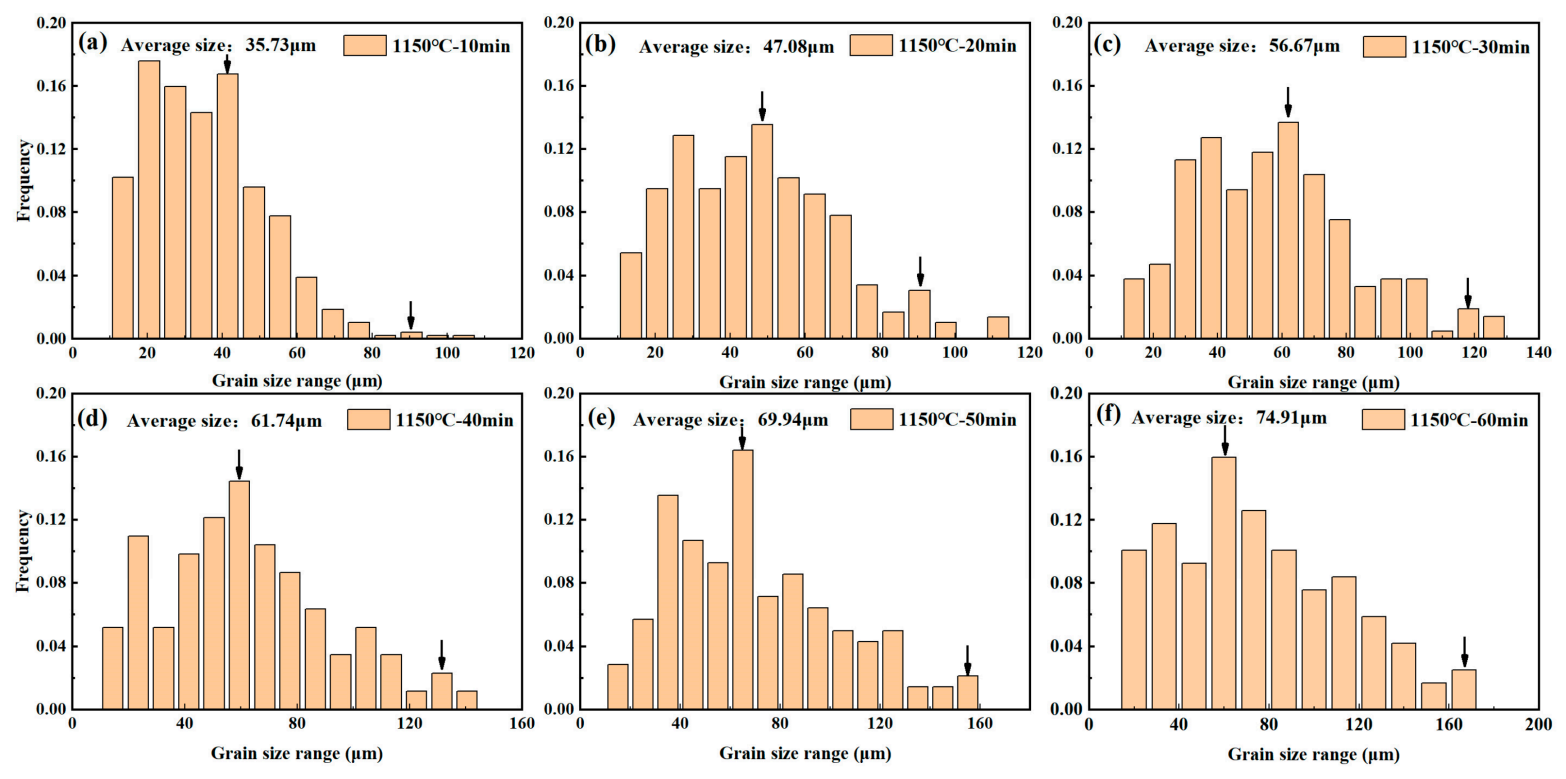
Simulated grain-size frequency distributions after different holding times at 1150 °C: (**a**) 10 min; (**b**) 20 min; (**c**) 30 min; (**d**) 40 min; (**e**) 50 min; (**f**) 60 min.

**Figure 11:**
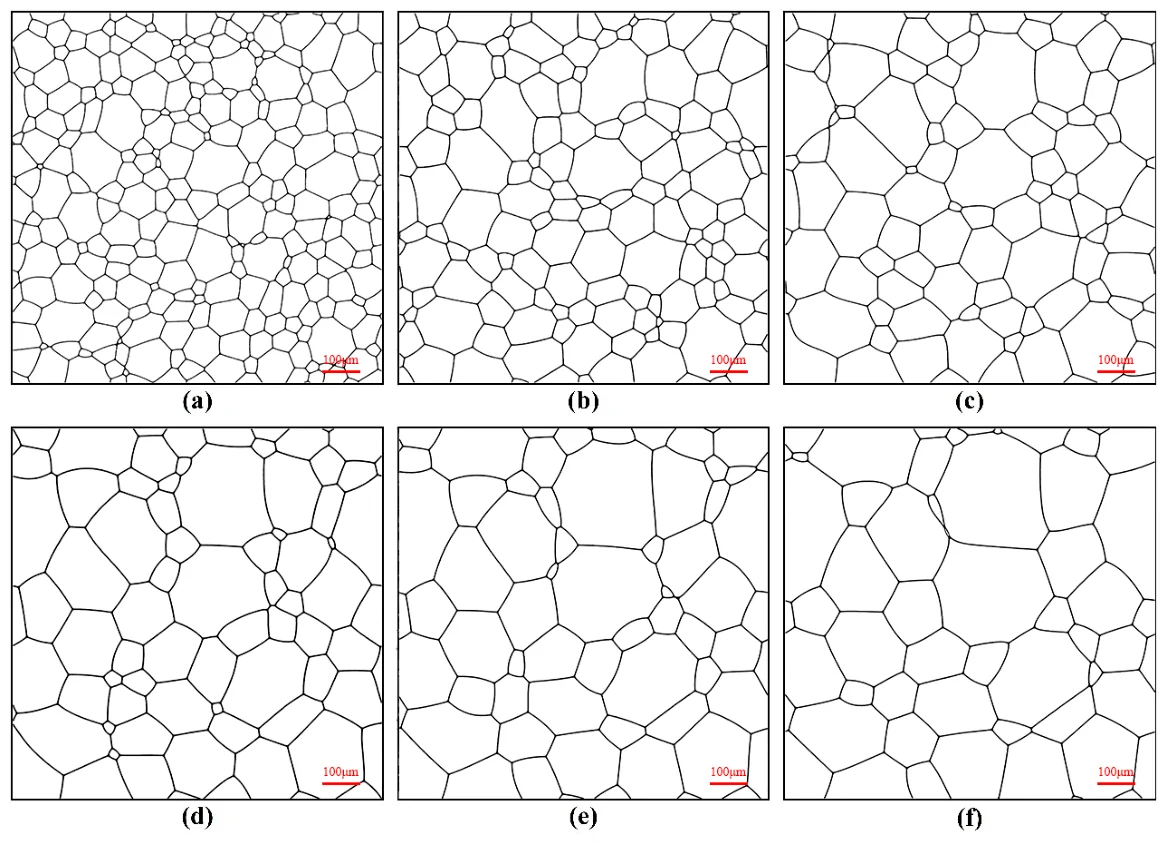
Evolution of the austenite grain structure during holding at 1300 °C: (**a**–**f**) holding times of 10, 20, 30, 40, 50, and 60 min, respectively.

**Figure 12:**
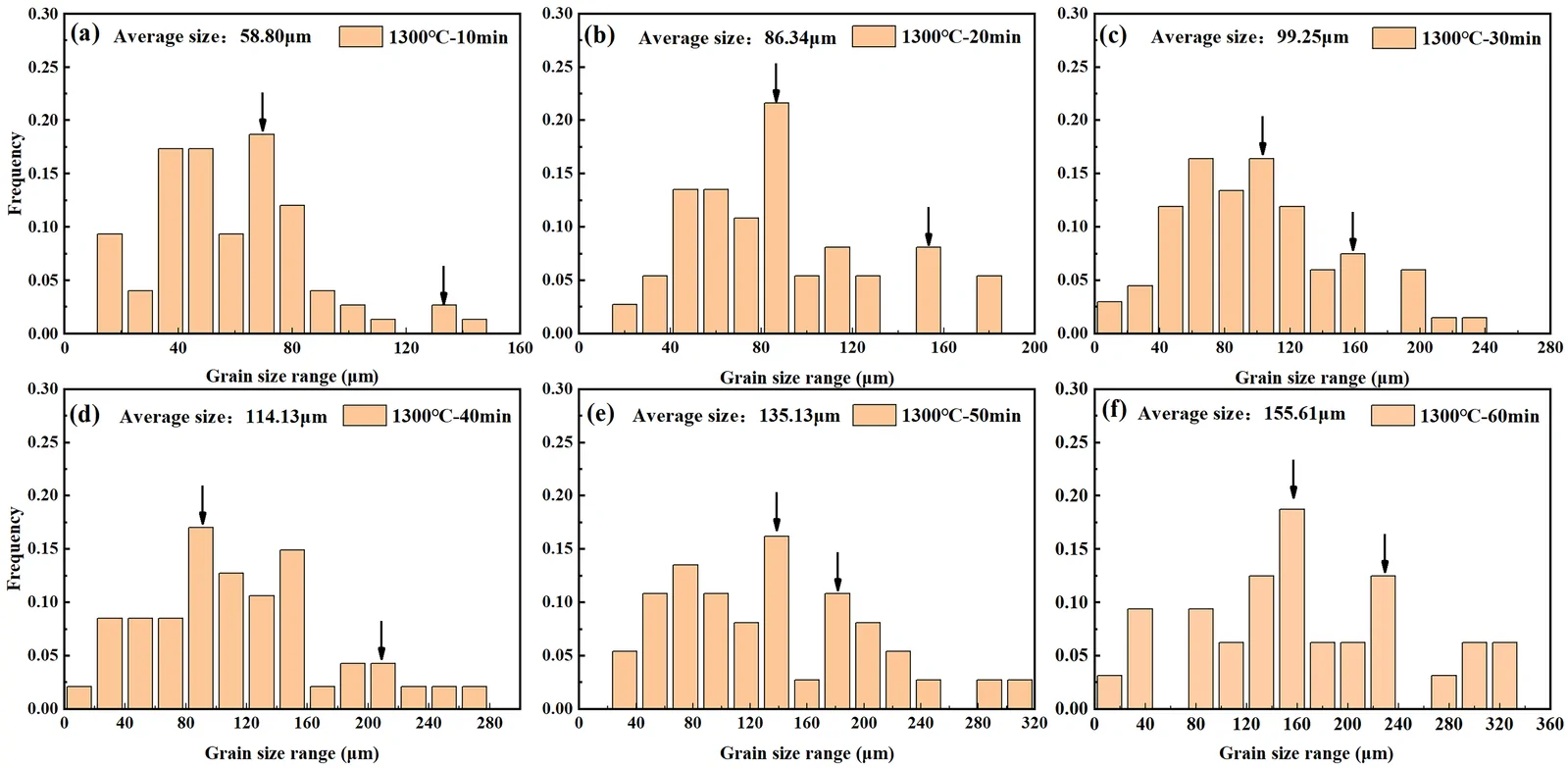
Simulated grain-size frequency distributions after different holding times at 1300 °C: (**a**) 10 min; (**b**) 20 min; (**c**) 30 min; (**d**) 40 min; (**e**) 50 min; (**f**) 60 min.

**Figure 13:**
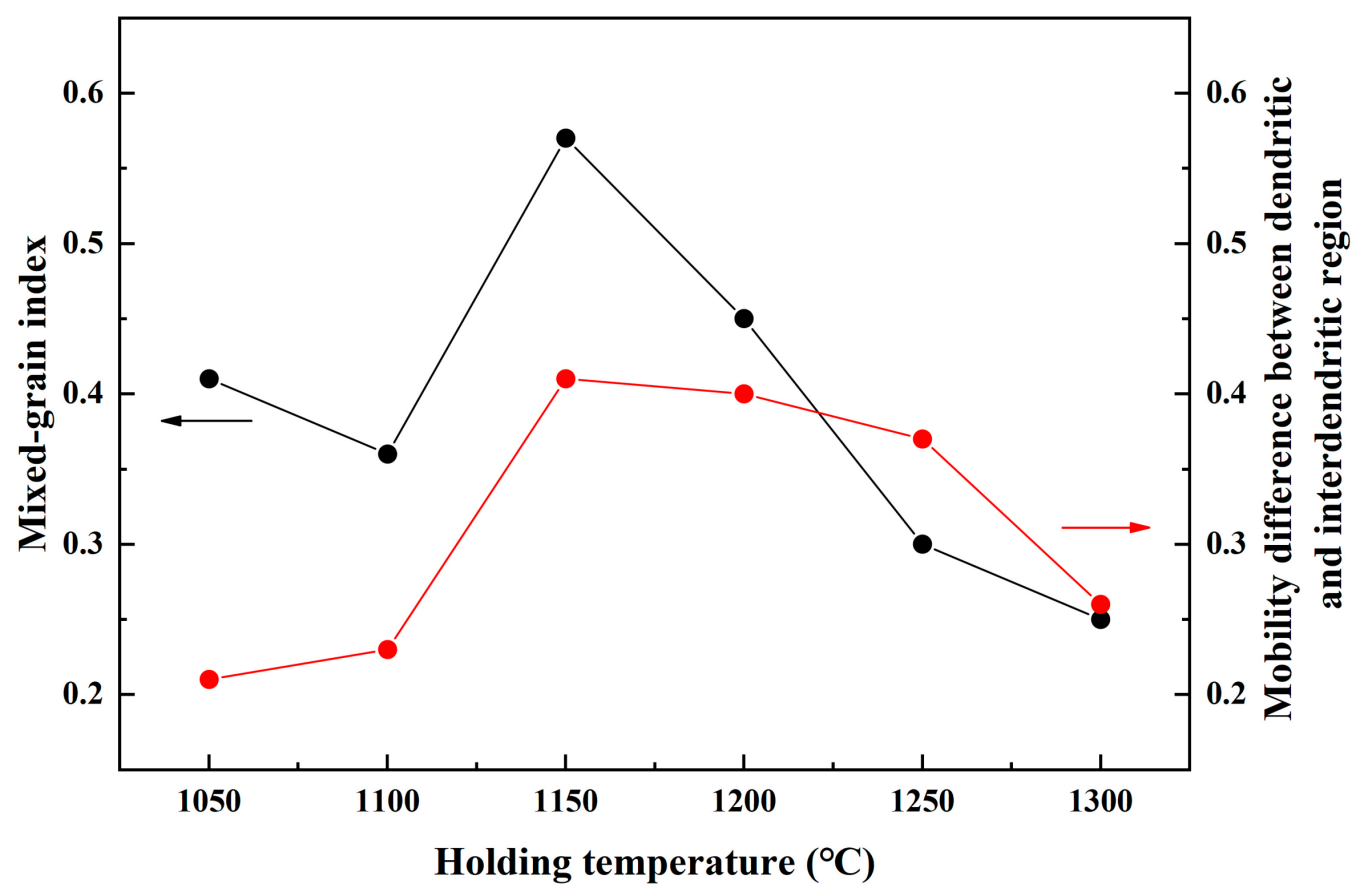
Mixed-grain index and relative difference in effective grain-boundary mobility after holding for 1 h at different temperatures.

**Table 1:** Chemical composition of the experimental steel (wt.%).

C	Si	Mn	P	S	Al	Ti	Nb	N
0.14	0.17	1.15	0.012	0.003	0.03	0.015	0.03	0.005

**Table 2:** Chemical compositions of dendritic and interdendritic regions (wt.%).

	C	Si	Mn	P	S	Al	Ti	Nb	N
Dendritic	0.07	0.14	1.02	0.007	0.001	0.025	0.007	0.012	0.003
Interdendritic	0.32	0.22	1.42	0.023	0.009	0.032	0.031	0.067	0.009

**Table 3:** Calculated grain-boundary mobility as a function of temperature.

Temperature (°C)	1050	1100	1150	1200	1250	1300
*M_GB_*	7.1 × 10^−5^	1.26 × 10^−4^	2.14 × 10^−4^	3.52 × 10^−4^	5.59 × 10^−4^	8.63 × 10^−4^

## Data Availability

The original contributions presented in this study are included in the article. Further inquiries can be directed to the corresponding authors.
